# The CRISPR-Cas system in *Lactiplantibacillus plantarum* strains: identification and characterization using a genome mining approach

**DOI:** 10.3389/fmicb.2024.1394756

**Published:** 2024-11-29

**Authors:** Mohaddeseh Rostampour, Bahman Panahi, Reza Masoumi Jahandizi

**Affiliations:** ^1^Department of Biology, University of Maragheh, Maragheh, Iran; ^2^Department of Genomics, Branch for Northwest and West Region, Agricultural Biotechnology Research Institute of Iran (ABRII), Agricultural Research, Education and Extension Organization (AREEO), Tabriz, Iran

**Keywords:** CRISPR-Cas, diversity, evolution, genome, phage

## Abstract

Clustered regularly interspaced short palindromic repeats (CRISPR) and CRISPR-associated (CAS) genes make up bacteria’s adaptive immune system against bacteriophages. In this study, 675 sequences of *Lactiplantibacillus plantarum* isolates deposited in GenBank were analyzed in terms of diversity, occurrence, and evolution of the CRISPR-Cas system. This study investigated the presence, structural variations, phylogenetic relationships, and diversity of CRISPR-Cas systems in 675 *L. plantarum* strains. The analysis revealed that 143 strains harbor confirmed CRISPR-Cas systems, with subtype II-A being predominant. Moreover, targeting phages and plasmid diversity between the predicted systems were dissected. The results indicated that approximately 22% of the isolates with verified and complete CRISPR systems exhibited the coexistence of both subtypes II-A and I-E within their genomes. The results further showed that in subtype II-A, the length of the repeat sequence was 36 nucleotides, on average. In addition, the number of spacers in subtypes II-A and I-E varied between 1–24 and 3–16 spacers, respectively. The results also indicated that subtype II-A has nine protospacer adjacent motifs, which are 5′-CC-3′, 5′-GAA-3′, 5′-TGG-3′, 5′-CTT-3′, 5′-GGG-3′, 5′-CAT-3′, 5′-CTC-3′, 5′-CCT-3′, and 5′-CGG-3′. In addition, the identified systems displayed a potential for targeting Lactobacillus phages. The investigation of the relationship between the targeting of Lactobacillus phages by the antiphage system in *L. plantarum* species showed that subtype II-A had the highest diversity in targeting Lactobacillus phages than subtype I-E. In conclusion, current findings offer a perspective on the prevalence and evolution of the CRISPR-Cas system in *L. plantarum*, contributing novel insights to the expanding field of CRISPR-Cas systems within lactobacillus strains. This knowledge establishes a foundation for future applied studies focused on enhancing phage resistance in industrial fermentation, reducing contamination risks, and improving product quality. The identified targeting diversity may also foster advancements in phage therapy through the development of CRISPR-based antimicrobials.

## Introduction

One of the most significant groups of microorganisms in the food industry is lactic acid bacteria (LAB) (Saad et al., 2013; Soleimani et al., 2023). LAB mainly produce lactic acid and different volatile metabolites during fermentation processes (Bintsis, 2018; Mazlumi et al., 2022; Sadeghi et al., 2022). In addition, probiotic functions are another characteristic of these bacteria used for improving the immunity of food products (Akoglu et al., 2015; Nami et al., 2020). Due to the ecological and metabolic adaptability of *L. plantarum*, it is widely found in fermented products such as sourdough, vegetables and fruits, cereals, and meat (Filannino et al., 2018; Manzoor and Tayyeb, 2019). These bacteria are frequently utilized as starters to enhance the flavor, texture, and organoleptic qualities of fermented food items due to their intriguing features (Cui et al., 2021).

Infection of LAB with bacteriophages is one of the critical challenges in the fermented food industry (Garneau and Moineau, 2011). Bacteriophages are a type of virus that infects only bacteria. In LAB, infection by bacteriophages leads to the destruction of host cells, slows down the fermentation process, and reduces the production of acid and aroma. Bacteriophages are predominantly resistant to pasteurization, and complete elimination of these viruses is usually a difficult process. Moreover, they can spread quickly and cause the destruction of the entire production chain, resulting in substantial economic damage. Nevertheless, some bacteria harbor adaptive defense mechanisms against phages known as the CRISPR/Cas (clustered regularly interspaced short palindromic repeats/CRISPR-associated protein) system in their genomes (Garneau et al., 2010). Using an “effector complex” made up of a CRISPR RNA (crRNA) guide, a group of Cas proteins (Class 1), or a single multi-domain Cas protein (Class 2), this system cleaves the target foreign invading DNAs and phages (Tautvydas et al., 2013; Chyou and Brown, 2019).

Classification of CRISPR systems is primarily based on the composition of the expression and interference modules of Cas genes in the respective CRISPR-Cas loci (Makarova et al., 2020). They are divided into two classes, six types, and three subtypes. Each CRISPR-associated protein comprises four functional modules: adaptation, expression, interference, and signal transduction. The analysis of encoded Cas genes within identified CRISPR-Cas systems unveils distinct characteristics of functional modules in each subtype. The adaptation module is composed of Cas1, Cas2, and Cas4. Cas1, a metal-dependent deoxyribonuclease, serves as an integrase during adaptation. Cas2 forms a tight complex with Cas1, while Cas4, a nuclease, cleaves single-stranded DNA (ssDNA) and contributes to protospacer processing and PAM identification (Koonin et al., 2017). The processing module, responsible for pre-crRNA processing, features the Cas9 protein. Our findings indicated that all identified subtypes, with the exception of the An197 strain, contain the Cas9 protein in their CRISPR systems. In subtype I-E, pre-crRNA processing is orchestrated by the Cas6 protein. The interference module, crucial for target recognition and cleavage, encompasses Cas3, Cas5–Cas8, Cas10, and Cas11 for class I, and Cas9, Cas12, or Cas13 for class II (Makarova et al., 2020). Cas3, a nuclease and helicase, is essential for interference. Notably, in subtype I-C, the role of Cas6 is performed by Cas5m. Cas8, another member of the interference module, is involved in PAM recognition (Khan et al., 2022). Genome-wide investigations of CRISPR-Cas systems are now possible due to recent developments in next-generation sequencing technology and the accessibility of many bacterial genomes. To reveal the underlying bacteria’s adaptive defense mechanism against phages, extensive studies have identified the CRISPR-Cas systems in different bacteria (Horvath et al., 2008; Held et al., 2010; van Belkum et al., 2015; Hidalgo-Cantabrana et al., 2017; Rossi et al., 2017; Ilıkkan, 2021; Yang et al., 2020; Nami et al., 2021; Rostampour et al., 2022). Recently, CRISPR loci frequency and diversity of targeting invasive DNA in the *Lactobacillus brevis* strains have been surveyed by Panahi et al. (2022) and Goh et al. (2023).

*Lactiplantibacillus plantarum* is one of the optional LAB that can tolerate high levels of acidity and ethanol concentration. It can also significantly increase microbiological stability and food safety. Among other noteworthy characteristics that differentiate *L. plantarum* from other LAB are its excellent compatibility with the fermentation process and its metabolic flexibility (Dinev et al., 2018; Prete et al., 2020). However, the features of the CRISPR-Cas system in *L. plantarum* are not well-understood. The occurrence of the CRISPR-Cas system in *L. plantarum* strains was dissected, and some information about the rate of occurrence of predicted CRISPR-Cas systems has been reported. It has been demonstrated that there is no CRISPR-Cas subtype III in *L. plantarum* strains (Crawley et al., 2018). However, the extensive functional diversity inherent in *L. plantarum* strains, stemming from the substantial genomic variation among strains, requires the isolation and sequencing of a large number of strains from diverse sources. Currently, public databases house a large number of whole-genome sequences of *L. plantarum* strains.

Gaining a holistic understanding of the occurrence, diversity, and functionality of CRISPR-Cas systems within this species provides an unprecedented chance for computational mining of these available genome sequences. This study aimed to perform a comprehensive genome mining analysis of 675 *L. plantarum* strains to uncover the occurrence, diversity, and characteristics of CRISPR-Cas systems. The findings will contribute to a deeper understanding of *L. plantarum*’s defense mechanisms and their potential applications in improving industrial fermentation processes.

## Materials and methods

### Genomics data selection and CRISPR-Cas system prediction

The National Center for Biotechnology Information (NCBI^1^) GenBank was searched for eligible data selection. Genomic sequences of 675 *L. plantarum* strains were selected and retrieved for further analysis. The identification of CRISPR sequences was conducted using CRISPRFinder2 (Grissa et al., 2007). This software detects direct repeat sequences ranging from 23 to 55 base pairs (bp). For a sequence to qualify as a “confirmed CRISPR,” CRISPRFinder2 requires at least three identical repeat regions in sequence and length, each separated by variable sequences. If only two repeats with a variable spacer are identified, the sequence is flagged as a “questionable CRISPR.” For our study, we only considered “confirmed CRISPRs” as determined by CRISPRFinder2 and searched for Cas genes adjacent to CRISPR loci. We uploaded complete nucleotide sequences of genomes in FASTA format to CRISPRFinder2, executing the software with default parameters. The identified CRISPR arrays, which include both repeat and spacer sequences, were saved for further analysis. For each identified CRISPR region, we examined 10 kb upstream and downstream for potential Cas genes. Adjacent genes and their protein sequences were analyzed using BLASTn and BLASTp against GenBank, with parameters set to an expectation threshold (e-value) of ≤0.01, a score greater than 40, and a maximum of 1,000 target sequences. We adjusted parameters to accommodate short sequences and tested various match/mismatch scores to capture sequences ranging from highly conserved to low conservation.

Protein sequence analyses were conducted using BLASTp against the non-redundant and reference protein databases, applying a significance threshold of e-value ≤0.01 and ≥80% sequence coverage. CRISPR arrays and Cas genes identified through CRISPRFinder2 were verified using CRISPROne^2^ (Zhang and Ye, 2017), where the nucleotide sequences of plasmids and chromosomes were processed with default settings to confirm the presence of CRISPR and associated Cas genes.

### Secondary structures of repeat sequence

Repeat sequences of confirmed CRISPR-Cas systems were extracted and made into a FASTA-formatted sequence file for all repeat pools using BioEdit software. The secondary structures of consensus repeat sequences of each type of CRISPR system were predicted using minimum free energy criteria by the RNA fold web server (Hofacker, 2003). Moreover, CRISPR loci were graphically presented, and the spacer/repeat array pattern was visualized using CRISPRVIZ tools (Nethery and Barrangou, 2019).

### Phylogeny analysis of identified CRISPR-Cas systems

For the Cas1 amino acid sequence, multiple sequence alignment was performed using MUSCLE version 3.6 with default parameters integrated into Molecular Evolutionary Genetics Analysis version 10 (MEGA7.0) software. Alignment gaps and missing data were removed using the complete deletion option. The resulting sequence alignments for each group were then used to construct phylogenetic trees with the neighbor-joining (NJ) method. Cas1 proteins related to CRISPR systems of *Lactobacillus brevis* (L.b) and *Lactobacillus johnsonii* (L.j) species were used in a comparative analysis to examine their relationships with identified CRISPR-Cas systems in *L. plantarum*.

### Identification of target phages and plasmids

To identify potential target phages and plasmids, we conducted similarity searches utilizing BLAST software under stringent criteria to ensure high specificity. Sequence alignments were evaluated, considering only hits with sequence similarities exceeding 85%, to ensure the selection of high-confidence matches. Furthermore, a maximum threshold of three mismatches was implemented to further refine the results, minimizing the inclusion of sequences with significant variability. This methodology facilitated the identification of phage and plasmid sequences closely related to the query sequence, emphasizing highly conserved regions and ensuring reliable target predictions for downstream analyses. To further refine the identification of target recognition motifs adjacent to CRISPR protospacer sequences, we utilized the CRISPR Target web server. For subtype II CRISPR systems, we analyzed the motifs flanking the 3′ end of protospacer sequences, while for subtype I systems, we assessed the adjacent motifs on the 5′ end. These motifs play critical roles in the identification and specificity of target sequences by serving as markers for CRISPR interference. Subsequent to motif detection, we visualized these flanking sequences using the WebLogo server, which provided graphical representations of sequence conservation across the target motifs. The outputs from WebLogo illustrated nucleotide frequency at each position within the motif, revealing patterns that are essential for understanding target sequence specificity and highlighting conserved bases likely involved in CRISPR-Cas interaction. This approach enabled us to identify and analyze key motifs adjacent to protospacers, which are crucial for effective CRISPR targeting and interference across different CRISPR-Cas subtypes (Crooks et al., 2004).

## Results and discussion

### Occurrence of the CRISPR-Cas systems

Extensive research has demonstrated the diverse prevalence and distribution of CRISPR-Cas systems across various bacterial taxa, underscoring their evolutionary significance and adaptive roles (Panahi et al., 2022; Nami et al., 2023; Goh et al., 2023; Panahi et al., 2023). In *L. plantarum*, recent analyses have illustrated the presence of distinct CRISPR-Cas subtypes across different strains, providing insights into lactic acid bacterial (LAB) immunity and genome evolution. This study examining 675 *L. plantarum* genomes from the NCBI database found that 122 strains (approximately 22%) possessed Cas genes, categorizing them as confirmed CRISPR-Cas carriers. Notably, a small portion of these strains (approximately 2%) exhibited both subtype II-A and I-E CRISPR-Cas systems, suggesting that certain strains may utilize multiple adaptive immune pathways for enhanced environmental resilience (Goh et al., 2023).

Subtype II-A has emerged as the most frequently occurring subtype in *L. plantarum*, with subtype I-E also present in several strains—a finding consistent across multiple LAB studies. This trend indicates that subtype II-A is the preferred CRISPR-Cas system for phage defense and genome stability, potentially due to its efficient spacer acquisition and robust interference processes (Yang et al., 2022; Schuster et al., 2019; Nami et al., 2023). Previous research has suggested that subtype II-A in LAB, particularly in Lactobacillus species, may have evolved as a primary defense mechanism, especially considering the challenges posed by bacteriophage exposure in fermentation and other high-risk environments (Horvath and Barrangou, 2010; Sorek et al., 2013).

The detection of dual subtypes (II-A and I-E) within individual genomes provides evidence of subtype coexistence, which may offer functional redundancy or enhanced adaptability under varying environmental pressures (Table 1). Strains with both subtypes may benefit from a broader defensive capacity, as each subtype possesses unique functional characteristics in spacer acquisition and targeting mechanisms. For example, subtype I-E differs in its reliance on protospacer adjacent motifs (PAMs) and may exhibit distinct targeting precision, which could complement the broader targeting range of subtype II-A (Makarova et al., 2020; Shmakov et al., 2017). This interplay between subtypes may be advantageous in niche environments where phage diversity and virulence are high, such as in dairy or gut microbiomes, where *L. plantarum* is commonly found.

**Table 1:** Clustered regularly interspaced short palindromic repeats/CRISPR-associated system in *L. plantarum* strains with confirmed CRISPR array.

Strains	CRISPR-Cas types	Direction	Total CRISPR	Strains	CRISPR Cas types	Total CRISPR	Direction
EGD-AQ4	II-A	+, −	2	HAC01	II-A	1	−
AG30	II-A	−	1	EBKLp545	II-A	2	−
ZS2058	II-A	+	1	SN35N	II-A	1	−
Nizo2801	II-A	+, −	2	E1	II-A	1	+
Nizo2029	II-A	+	1	DR7	II-A	1	−
LZ206	II-A	−	2	JMCC0013	II-A	2	+
LZ227	II-A	−	1	FUA3428	II-A	2	+
MPL16	II-A	−	2	dkp1	II-A	1	+
LY-78	II-A	−	1	Curd	II-A	1	+
JSA22	II-A	+	1	R77	II-A	2	+
MF1298	II-A	+, −	2	FUA3302	II-A	3	+, +, −
MHO2.5	II-A	+	1	DS6_9	II-A	2	+, −
MHO2.9	II-A	+, −	2	LR46	II-A	1	+
RI-146	II-A	+, −	2	B1.1	II-A	2	+
RI-514	II-A	−	1	R47	II-A	2	−
RI-513	II-A	−	1	ZFM4	II-A	1	−
CLP0611	II-A	+	1	WS1.1	II-A	1	−
Lp790	II-A	+, −	2	Dm-2019-33	II-A	1	+
CGMCC 1.557	II-A	+	1	LR39	II-A	2	+
SF 15C	II-A	+	1	LR14	II-A	1	−
RI-265	II-A	−	1	R75	II-A	2	−
RI-029	II-A	+, −	2	7.8.4	II-A	1	−
LMG S-29189	II-A	+	1	R58	II-A	2	−
CECT 8962	II-A	+	1	R105	II-A	2	+
CECT 8963	II-A	+, −	2	KACC92189	II-A	1	−
CECT 8964	II-A	+, −	2	FBL-3a	II-A	1	−
CECT 8965	II-A	+, −	2	SRCM101167	II-A	1	−
CECT 8966	II-A	+, −	2	ATG-K8	II-A	1	−
K25	II-A	+	1	DW12	II-A	2	+
JDARSH	II-A	−	1	SRCM101518	II-A	1	−
DS11_9	II-A	+	1	QS7	II-A	1	+
DS9_9	II-A	+	1	YT041	II-A	1	−
DS14_9	II-A	−	1	JZ6	II-A	1	+
DS18_9	II-A	−	1	LB356R	II-A	1	−
DS23_9	II-A	−	1	LRCC5310	II-A	1	+
LQ80	II-A	+, −	2	DSM 8862	II-A	1	+
IRG1	II-A	+	2	R35	II-A	1	+
CECT 9434	II-A	+	1	R106	II-A	1	−
CECT 9435	II-A	+	1	R49	II-A	1	+
FUA3590	II-A	−	2	R102	II-A	1	−
UNQLp11	II-A	−	1	R98	II-A	1	−
J26	II-A	−	1	R46	II-A	1	−
R62	II-A	+	1	022AE	II-A	1	+
R95	II-A	+	1	P9	II-A	1	+
Dm-2019-48	II-A	+	1	RI-191	II-A	1	+
M19	II-A	+	1	DSM 8866	II-A	1	+
MCC636	II-A	+	1	LPG8	I-E	1	+
2025	II-A	−	1	RI-165	I-E	1	+
3.2.8	II-A	+, −	2	CIF17AN2	I-E	1	−
EGD-AQ4	I-E	+	1	CIF17A2	I-E	1	−
SF2A35B	I-E	+	1	CIF17A5	I-E	1	+
Nizo1839	I-E	−	1	CIF17AN8	I-E	1	−
MPL16	I-E	+	1	CIF17A4	I-E	1	+

The 22% prevalence of CRISPR-Cas systems in *L. plantarum* strains should also be considered. This frequency is somewhat lower than that observed in other bacterial species, raising questions regarding alternative defense strategies within *L. plantarum*. Strains lacking CRISPR-Cas systems may employ alternative mechanisms, such as restriction-modification systems, abortive infection strategies, or the production of bacteriocins, which can provide resistance to phages in the absence of CRISPR-Cas (Canchaya et al., 2003; Labrie and Moineau, 2010). These systems may be particularly adaptive in environments where CRISPR-Cas-mediated immunity is either redundant or less effective against certain phage populations.

Consistent with other studies, our findings reaffirm that CRISPR-Cas subtypes II-A and I-E are conserved in lactic acid bacteria (LAB), highlighting their evolutionary importance and adaptive functionality in these organisms (Yang et al., 2022; Schuster et al., 2019; Nami et al., 2023). This conserved presence supports the subtype-specific functionality in LAB immunity and underscores the potential role of CRISPR-Cas systems in enhancing the industrial applications of LAB strains. In microbial biotechnology, understanding CRISPR-Cas diversity among LAB could refine strategies for strain selection, phage resistance, and genome editing applications, as different subtypes may offer distinct advantages in these efforts (Barrangou and Horvath, 2012). Further studies could explore the selective pressures driving the predominance of these subtypes, examining how CRISPR-Cas diversity affects strain performance under industrial conditions. In addition, research into the ecological roles of each subtype could elucidate how CRISPR-Cas systems impact LAB survival, adaptation, and horizontal gene transfer, particularly in high-competition environments such as the human gut and fermented foods.

### Diversity of the CRISPR-Cas system

**Figure 1:**
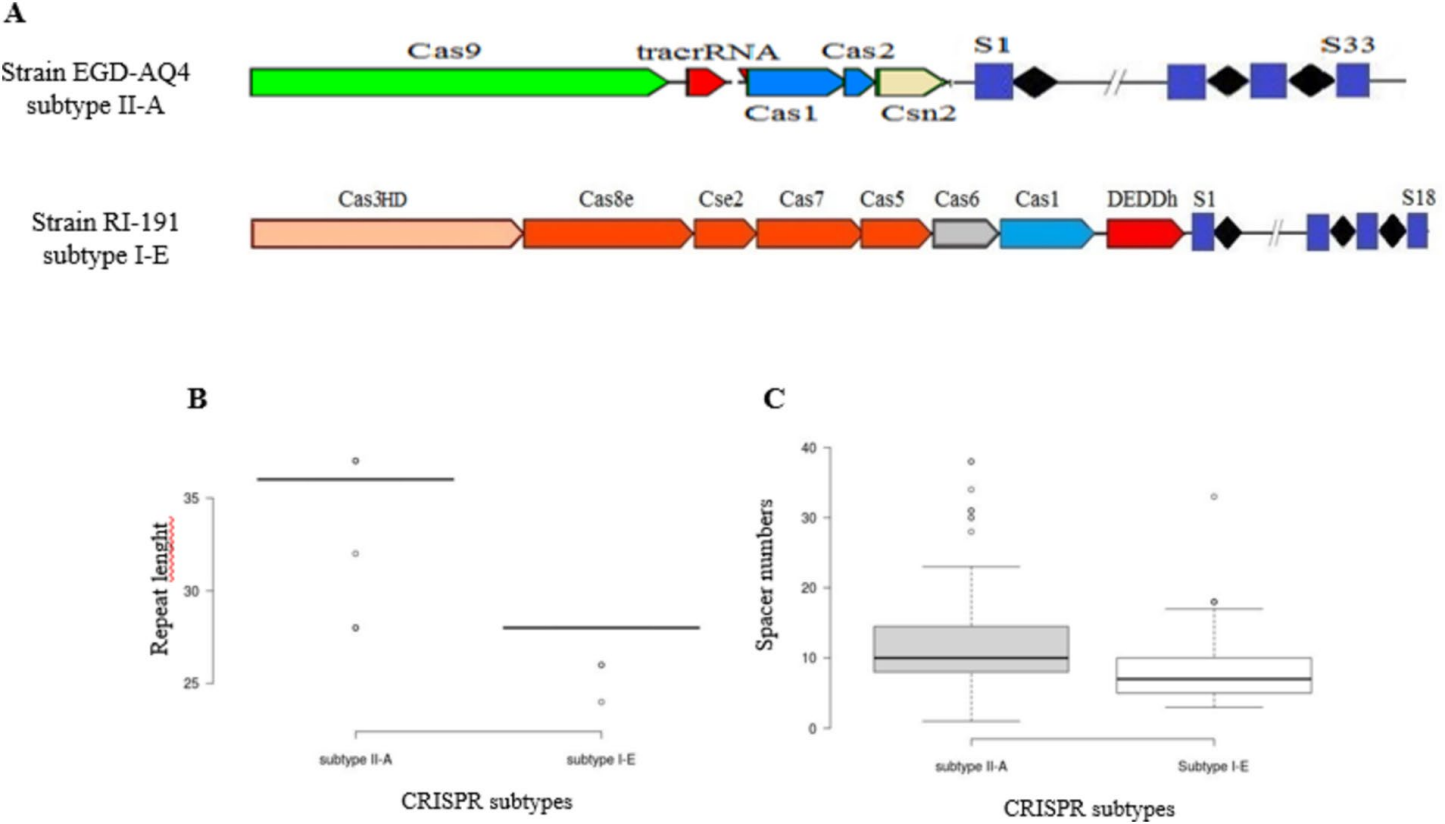
Schematic diagram of structural variation of predicted CRISPR-Cas array **(A)**. Variation in the length of conserved repeats **(B)** and distribution of spacer numbers for each CRISPR-Cas subtype **(C)**.

The data from Figure 1A reveal subtype-specific distinctions among *L. plantarum* strains, with 125 strains exhibiting subtype II-A and 18 strains exhibiting subtype I-E among those with fully functional CRISPR-Cas systems. These subtypes differ in prevalence, encoded components, and structural organization, which may contribute to their varied roles and efficiencies in immunity. For instance, subtype II-A in *L. plantarum* is characterized by the presence of Cas9, Cas1, Cas2, and the Csn2 genes, an arrangement believed to enhance precision in spacer acquisition and interference (Goh et al., 2023). This subtype’s signature protein, Cas9, is known for its sequence-specific DNA targeting ability, rendering subtype II-A highly effective in adaptive immunity, particularly against diverse bacteriophages (Sorek et al., 2013). In contrast, subtype I-E is marked by a multi-protein system, including Cas3HD and components such as Cas8e, Cse2, Cas7, and Deddh, which rely on the concerted activity of these proteins to form interference complexes. This structure is common in I-E subtypes, where Cas3’s nuclease activity is critical to DNA degradation, but may entail different interference efficiencies compared to Cas9-based subtypes (Makarova et al., 2020).

An examination of conserved repeat lengths in *L. plantarum* revealed further subtype-specific structural nuances. Subtype II-A exhibits repeat lengths ranging from 28 to 36 nucleotides, with an average of 36 nucleotides, while subtype I-E has shorter repeats averaging approximately 28 nucleotides (Figure 1B). These variations in repeat length may reflect evolutionary adjustments to ensure stability and functionality within each subtype as longer repeats in II-A may contribute to a more stable repeat-spacer array (Panahi et al., 2022). Such subtype-specific repeat lengths and spacer configurations, also corroborated by studies in *Lactobacillus johnsonii* and *L. casei*, underscore the adaptive significance of these differences (Nami et al., 2023; Ghaffarian and Panahi, 2023).

Notably, the number of spacers varied significantly between subtypes, ranging from 1 to 24 in subtype II-A and 3–16 in subtype I-E, indicating that subtype II-A likely plays a more active role in *L. plantarum* defense due to its larger repeat-spacer arrays (Figure 1C). The extensive spacer diversity observed in II-A may suggest a higher phage exposure or evolutionary selection for adaptive flexibility in environments such as food fermentation and the gut microbiome, where *L. plantarum* frequently resides (Horvath and Barrangou, 2010). The larger spacer array capacity in subtype II-A is thought to correlate with enhanced adaptation, potentially providing *L. plantarum* with broader or more effective immune memory, especially in dynamic microbial communities (Shmakov et al., 2017).

The predominance of subtype II-A in *L. plantarum* highlights differences from other Lactobacillus species, where subtype I-E is often more common. For instance, studies in *L. johnsonii* and *L. casei* suggest that I-E prevalence may relate to ecological niches or specific viral exposures favoring subtype I-E’s multi-protein interference complexes (Nami et al., 2023; Yang et al., 2020). The presence of more complex I-E systems in these species may reflect distinct requirements for phage defense in these LAB environments, where multi-protein complexes, such as those in subtype I-E, may provide functional advantages (Makarova et al., 2020).

**Figure 2:**
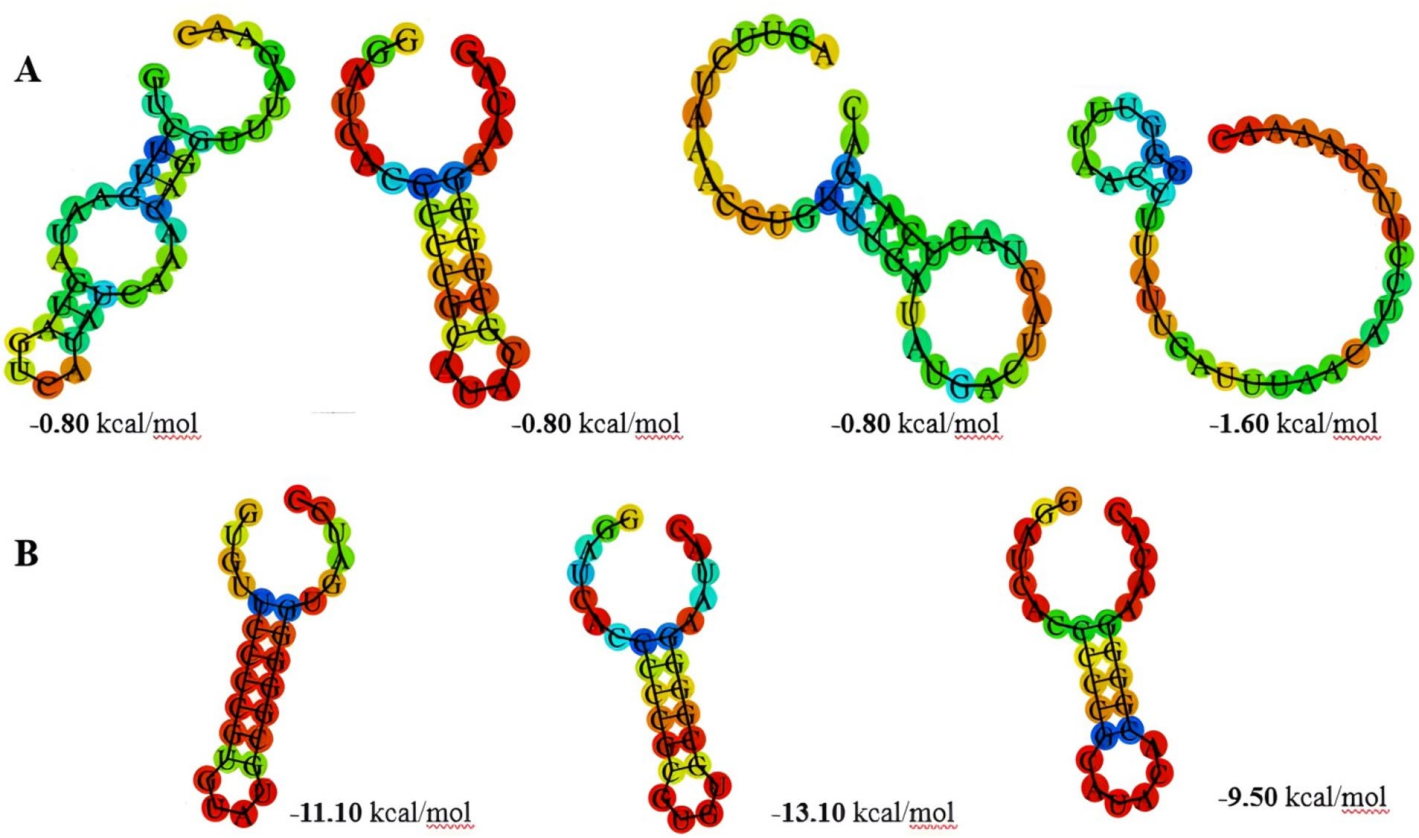
The prediction of consensus direct repeat secondary structure and corresponding MFE values in subtype II-A **(A)** and subtype I-E **(B)**.

The findings indicated notable differences between subtypes II-A and I-E regarding their direct repeat structures, which are crucial for the system’s overall stability and efficiency. Subtype II-A exhibited the shortest stem and lowest minimum free energy (MFE) values (Figure 2A), suggesting a less stable secondary structure compared to subtype I-E. The stability of RNA structures, particularly in CRISPR systems, plays a significant role in the processing of CRISPR RNA (crRNA) and the efficacy of the interference mechanism. Lower MFE values in subtype II-A may imply that these repeat sequences are more flexible and less energetically favorable in their secondary structure, potentially affecting their interaction with Cas proteins during crRNA maturation and interference processes (Zhang et al., 2020). The dynamic nature of these structures may allow for rapid adaptation in response to fluctuating environmental pressures, such as the presence of phages. In contrast, subtype I-E exhibited longer stem lengths and higher Guanidine and Cytosine (GC) content, contributing to the formation of more stable structures (Figure 2B). The stability conferred by these structural features is likely beneficial for the integrity of the CRISPR-Cas system, allowing for more efficient crRNA processing and a robust immune response against invading genetic elements. Higher GC content typically enhances thermal stability, making the RNA structures less susceptible to degradation and potentially improving the longevity of the immune memory (Bult et al., 1996). This greater stability may facilitate consistent phage recognition and degradation, providing a selective advantage in environments where phage pressure is high, as observed in many fermentation processes. These structural variations align with previous findings in other Lactobacillus species, such as *L. casei*, *L. brevis*, and *L. johnsonii*, where similar trends in CRISPR repeat structure and stability have been reported (Yang et al., 2020; Panahi et al., 2022; Nami et al., 2023). Such consistency across different Lactobacillus species suggests a conserved evolutionary strategy in which the structural attributes of CRISPR systems are tuned to optimize the immune response according to the specific ecological niches these bacteria occupy.

**Figure 3:**
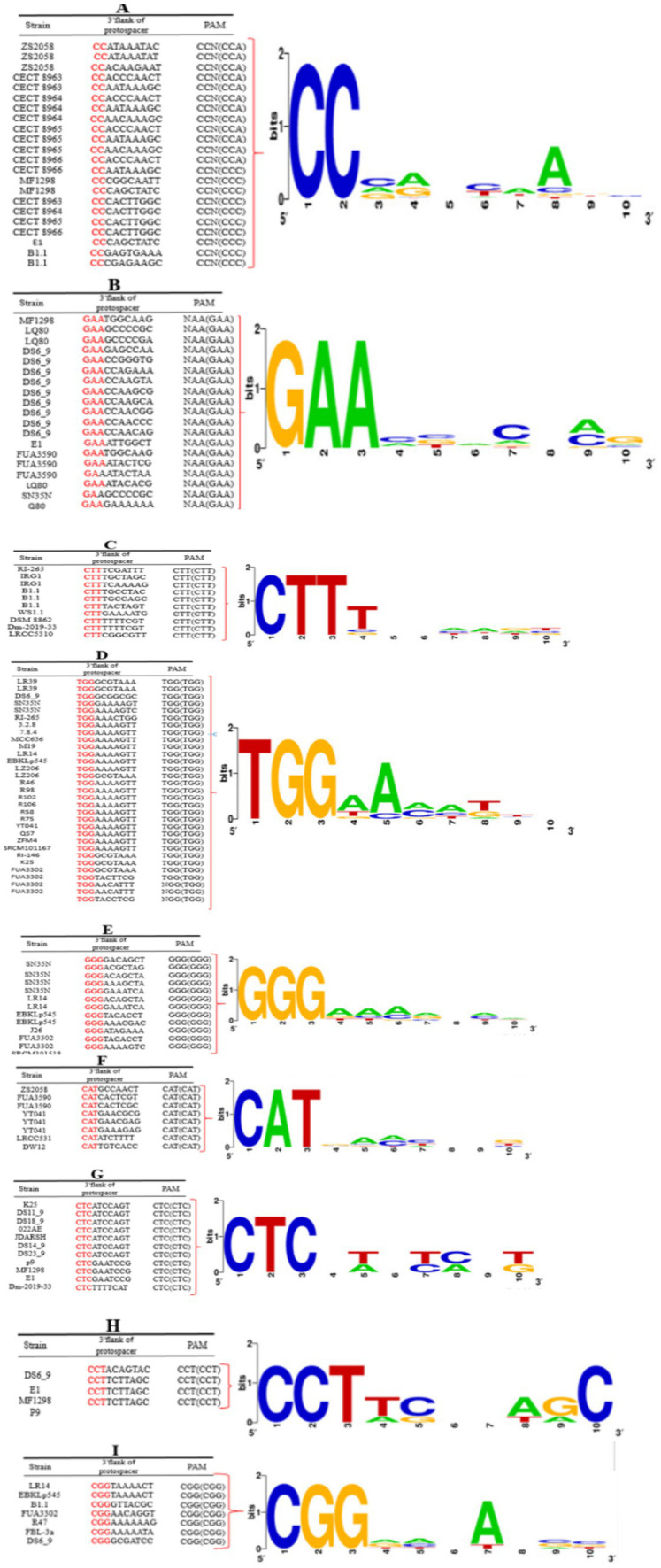
Predicted PAM motifs in *L. plantarum* strains. The height of each letter represents the conservation of that nucleotide at each position. PAM motifs were extracted from the 10-nt flank at the 3′ end for subtypes II-A **(A–H)** and from the 10-nt flank on the 5′ end for subtype I-E **(I)** of the predicted protospacers. Red-highlighted nucleotides in the given sequences are the consensus sequence of predicted PAM motifs in each strain.

The protospacer adjacent motif (PAM) is a critical component of CRISPR-Cas systems, functioning as a recognition signal that facilitates the binding of Cas proteins to foreign nucleic acids, such as those from invading phages or plasmids. PAMs are typically short, conserved sequences ranging from two to six base pairs that flank the target sequence in the invading DNA (Mojica et al., 2009; Shah et al., 2013; Panahi et al., 2022). The specificity and efficiency of the CRISPR-Cas immune response depend on the presence and identity of these PAMs, allowing Cas proteins to distinguish self from non-self DNA. In the current study, we identified nine distinct PAM motifs associated with the CRISPR-Cas subtype II-A: 5′-CC-3′, 5′-GAA-3′, 5′-TGG-3′, 5′-CTT-3′, 5′-GGG-3′, 5′-CAT-3′, 5′-CTC-3′, 5′-CCT-3′, and 5′-CGG-3′ (Figure 3). The diversity of PAM sequences in subtype II-A indicates a potential for recognizing a wide array of target sequences, enhancing the adaptability of these strains in combating various phage infections. The identification of specific PAM sequences in subtype II-A is significant for understanding the mechanics of CRISPR-Cas immunity in *L. plantarum*. Each PAM sequence likely plays a role in the specificity of target recognition, influencing which phages or plasmids can be effectively targeted by the CRISPR-Cas system. This specificity is crucial for the successful elimination of potential threats while minimizing collateral damage to the host’s genomic integrity. Such precision is especially important in industrial applications, where maintaining the viability of beneficial strains is paramount.

In contrast, the absence of a defined PAM for the CRISPR-Cas subtype I-E system in our study raises questions about the functionality and potential limitations of this subtype. While subtype I-E systems are generally thought to rely on different recognition mechanisms, the lack of identified PAMs could indicate a reduced capability for efficient target recognition and interference compared to subtype II-A. This distinction may reflect the evolutionary pressures faced by these subtypes within different ecological contexts, where the adaptability of the CRISPR-Cas system can significantly influence the survival of the bacteria.

### Phylogenetic analyses

**Figure 4:**
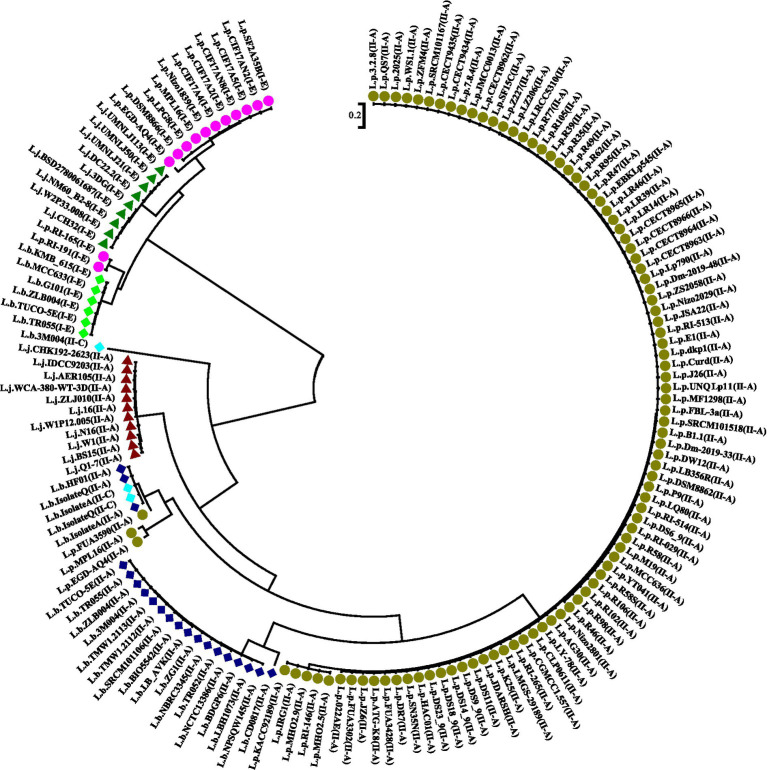
Phylogeny tree based on Cas1 amino acid sequence in subtypes II-A, I-E, and II-C, belonging to *L. plantarum*, *L. johnsonii*, and *L. brevis.* Dark green and purple circles indicate the subtypes II-A and I-E in *L. plantarum* strains, respectively. Dark blue, light green, and light blue indicate the CRSPR-Cas system subtypes II-A, I-E, and II-C in *L. brevis*, respectively. Green and red triangle shapes also indicate the subtypes I-E and II-A in *L. johnsonii*, respectively.

The analysis of Cas1 sequences through multiple alignments and phylogenetic approaches is crucial for understanding the evolutionary dynamics of CRISPR-Cas systems. As illustrated in Figure 4, our phylogenetic analysis revealed that subtype II-A CRISPR-Cas systems in *L. plantarum* (L.p), *Lactobacillus brevis* (L.b), and *Lactobacillus johnsonii* (L.j) are organized into distinct clades. This clustering indicates significant divergence in the amino acid sequences of Cas1 among these species, underscoring the unique evolutionary trajectories shaped by various selective pressures and mutations within their respective environments.

The divergence observed in the Cas1 sequences reinforces the notion that CRISPR-Cas systems are not merely conserved genetic elements but dynamic entities that evolve in response to ecological demands. The findings support the studies of Panahi et al. (2022) and Nami et al. (2023), which identified strain-level variations in the fundamental components of CRISPR-Cas systems. These studies emphasize that even within closely related species, CRISPR-Cas systems can exhibit significant evolutionary divergence, potentially due to different interactions with phages and plasmids in their specific habitats. In a broader context, our phylogenetic results are consistent with findings from other bacterial genera, such as Clostridium, where distinct subgroups also displayed unique evolutionary pathways (Long et al., 2019). This pattern suggests that CRISPR-Cas systems across various taxa may adapt to their environments in ways that are both species-specific and influenced by the prevailing microbial communities. Such evolutionary adaptations are likely driven by the need for effective defense mechanisms against diverse phage populations, which can vary significantly even among closely related strains.

### Targeting diversity of predicted CRISPR-Cas systems

**Figure 5:**
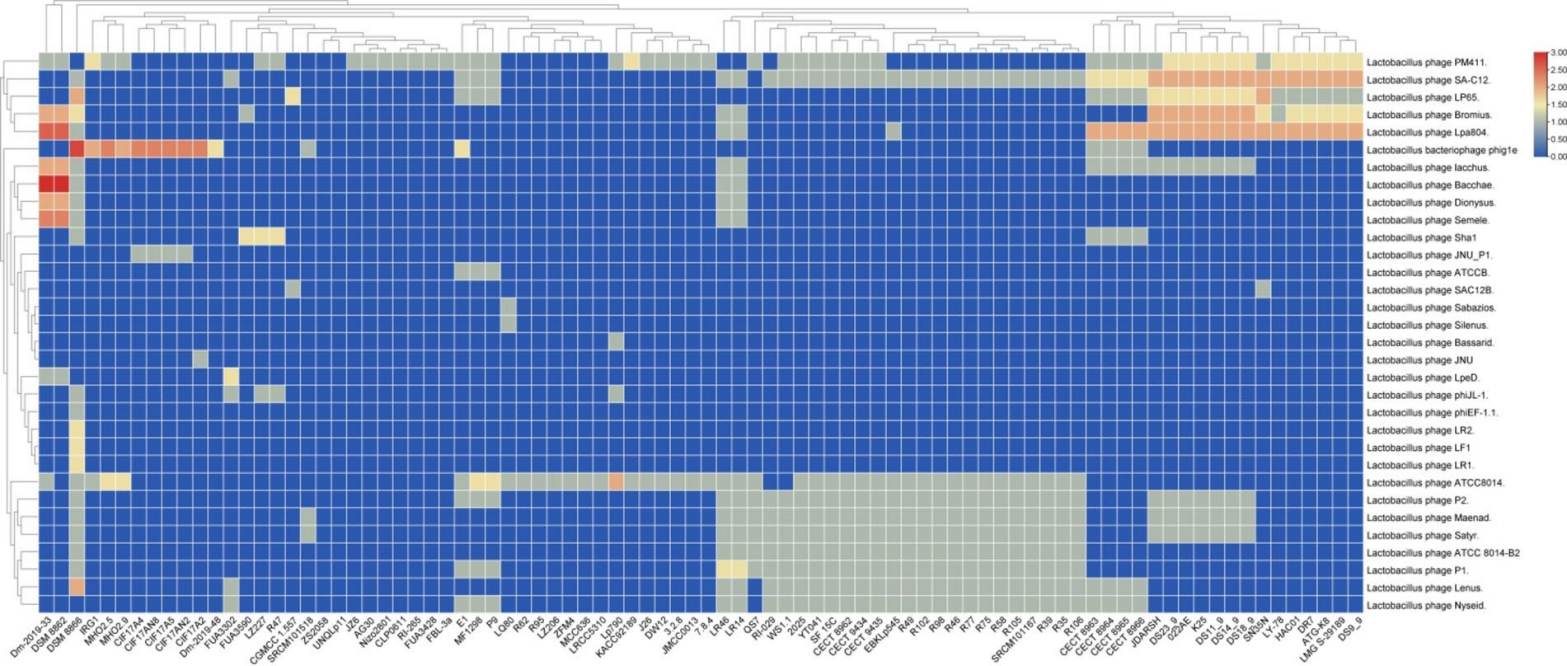
Targeting phage diversity by the predicted CRISPR-Cas systems in *L. plantarum* strains. Each box shows the presence of the target phages, and the color range indicates the frequency of corresponding targets. Blue and red indicate the low number and the high number, respectively. Blue and red indicate the low number and the high number, respectively.

To gain deeper insights into the complexity and efficacy of the CRISPR systems in the examined *L. plantarum* strains, a similarity analysis of spacer sequences against known phage and plasmid sequences is essential. This study identified the primary Lactobacillus phages targeted by the antiphage systems present in *L. plantarum* (Figure 5). Our findings indicated that subtype II-A exhibited the highest diversity in targeting Lactobacillus phages compared to subtype I-E (Figure 5). This observation aligns with previous reports that underscore the predominance of subtype II-A CRISPR-Cas systems in targeting a broad array of Lactobacillus phages (Goh et al., 2023).

The diversity in targeting suggests that subtype II-A may possess a broader range of protective capabilities against phage attacks, which is crucial for maintaining the viability of these strains in competitive environments. This adaptability could be attributed to the structural and functional characteristics of the CRISPR-Cas systems, which may facilitate the recognition of diverse phage genomes. Understanding the mechanisms underlying this diversity can enhance our knowledge of bacterial immunity and potentially guide the development of phage-resistant bacterial strains for industrial applications.

**Figure 6:**
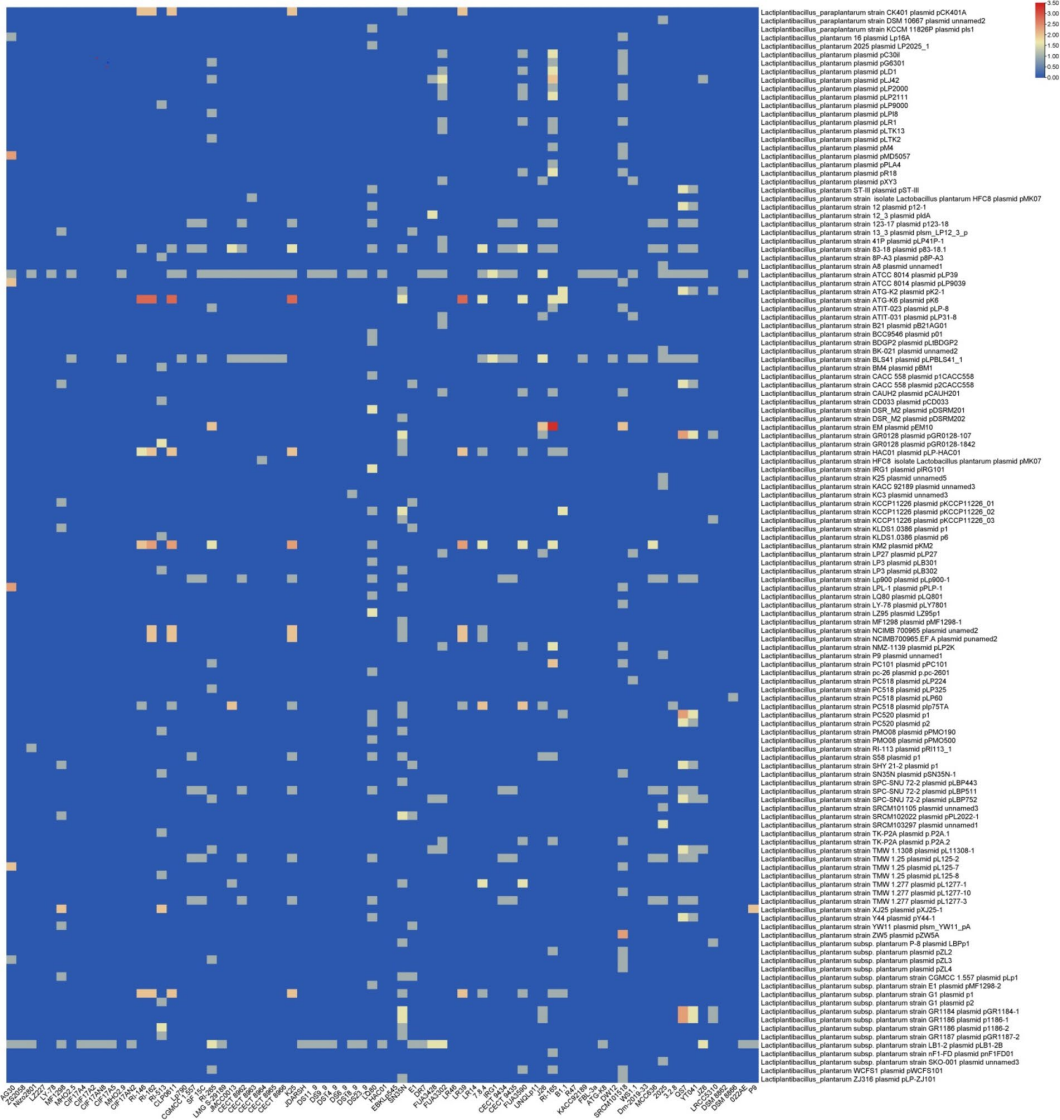
Targeting plasmid diversity by the predicted CRISPR-Cas systems in *L. plantarum* strains. Each box shows the presence of the target plasmid, and the color range indicates the frequency of corresponding targets. Blue and red indicate the low number and the high number, respectively.

Moreover, homology analysis of the spacer sequences from the identified CRISPR-Cas systems in *L. plantarum* showed that these systems predominantly target plasmids from other Lactobacillus species, such as Lacticaseibacillus (Paracasei subspecies), Lactococcus (Lactis subspecies), and Pediococcus (Figure 6). These findings are partially consistent with previous research conducted by Goh et al. (2023), which examined the characteristics of CRISPR-Cas systems in *L. brevis*. The homology between spacer sequences and plasmid sequences indicates that *L. plantarum* has evolved mechanisms to defend against not only phage infections but also plasmid-mediated gene transfer, potentially conferring competitive advantages in natural environments.

The targeting diversity of the identified CRISPR-Cas systems against plasmids revealed that three strains with subtype II-A exhibited greater diversity in targeting *L. plantarum* plasmids. In contrast, only two strains with subtype I-E, specifically RI-165 and DSM8866, exhibited varying levels of targeting diversity (Figure 6). This pattern aligns with previous findings in *L. brevis*, which reported that subtype II-A possesses a greater capacity for targeting *L. plantarum* plasmids compared to subtype I-E (Panahi et al., 2022). The implications of this targeting capability suggested that subtype II-A may play a critical role in maintaining genomic stability and preventing unwanted genetic elements that could disrupt the metabolic processes of *L. plantarum*.

Regarding plasmids from *Lacticaseibacillus casei.*, five strains with subtype II-A also exhibited greater diversity in targeting these plasmids. This further underscores the versatility of subtype II-A systems, which can adapt to recognize and target a variety of plasmid types from different genera. Such targeting diversity may enhance the ecological fitness of *L. plantarum* by enabling it to navigate complex microbial ecosystems, where plasmid transfer is a common phenomenon.

### Spacerosome analysis

**Figure 7:**
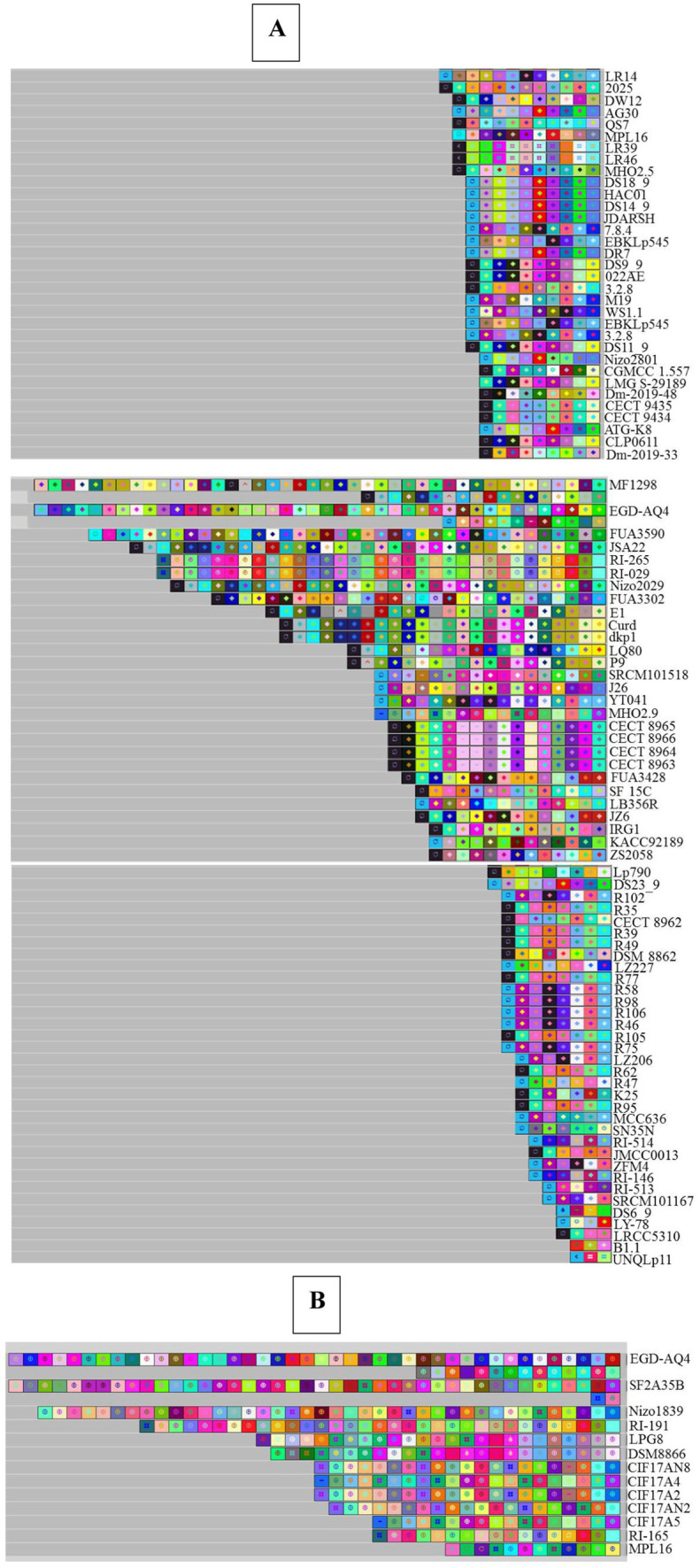
Graphical representation of spacer pattern in subtypes II-A **(A)** and I-E **(B)** CRISPR-Cas systems. Gray squares containing an “X” represent no spacers. Each color combination represents a unique spacer sequence based on the nucleotide sequences. The newly and earliest acquired spacers are shown on the left and right sides, respectively.

Based on the analysis of the acquisition and deletion of spacers under the selection pressure of foreign invading DNA, the evolutionary paths of the discovered systems in *L. plantarum* strains were determined. Based on the nucleotide sequences, each color composition indicated a different spacer sequence, and the spacers were sorted from the ancestral end toward the recently acquired end (left) (Panahi et al., 2022; Nami et al., 2023). In addition, depending on the nucleotide sequences, each color composition displayed a unique spacer sequence. According to the data shown in Figure 7, it is clear that the spacers for CECT 8963, CECT 8964, CECT 8965, and CECT 8966 consistently had an identical pattern of composition, acquisition, and deletion during the evolutionary era, but other strains had distinct spacers that were subsequently acquired. Strains with the same spacing pattern may have a common ancestor. Based on this hypothesis, R35, R39, R49, R77, R105, R62, and R95 originate from common lineages (Figure 7A).

Figure 7B depicts an analysis of spacer combination and acquisition in the spacer of the subtype I-E CRISPR-Cas system. The spacer alignment in the strain with subtype I-E displayed a distinctive pattern, showing that the strains had diverse later obtained spacers. Among the strains that contain the subtype I-E CRISPR/Cas systems—CIF17AN8 and CIF17A2, isolated from feces—showed a similar evolutionary pattern in terms of composition, acquisition, and deletion of spacers in their evolutionary period and referred that these strains originated from a shared ancestor (Long et al., 2019).

## Conclusion

In summary, the investigation into CRISPR-Cas systems within *L. plantarum* unveiled a diverse landscape with 143 strains featuring confirmed systems. Approximately 22% of these strains exhibited verified CRISPR arrays, while the rest displayed orphan CRISPR-Cas systems and were excluded from the analysis. The prevalent subtypes were identified as II-A and I-E, aligning with previous research on lactic acid bacteria. Interestingly, certain isolates showed the coexistence of subtypes II-A and I-E in their genomes, emphasizing the complexity of CRISPR-Cas configurations within *L. plantarum*. Structural analyses of conserved repeats revealed variations in lengths, shedding light on the diversity within these systems. Subtype II-A exhibited a higher prevalence compared to subtype I-E. Further exploration into conserved repeat secondary structures highlighted distinctive characteristics between subtypes, with subtype II-A showing shorter stems and lower minimum free energy values than subtype I-E. Phylogenetic analyses based on Cas1 sequences underscored evolutionary divergence among CRISPR-Cas systems in *L. plantarum*, *L. johnsonii*, and *L. brevis*, with separate clades for subtypes II-A and I-E within *L. plantarum*, suggesting unique evolutionary paths at the amino acid sequence level. The study expanded our understanding of CRISPR-Cas systems by delving into the functional elements, particularly the protospacer adjacent motif (PAM). Exploring the diversity of CRISPR-Cas systems’ targets revealed a higher diversity in phage targeting for subtype II-A compared to I-E. The investigation into spacer acquisition and deletion provided insights into the evolutionary trajectories of CRISPR-Cas systems in specific strains, suggesting common ancestry among strains with similar spacer patterns. Overall, this research contributes valuable information on the complexity, diversity, and evolutionary dynamics of CRISPR-Cas systems in *L. plantarum*.

## Data Availability

The data presented in the study are deposited in the NCBI with different accessions provided in Supplementary material.

## Footnotes

^1^https://www.ncbi.nlm.nih.gov/

^2^https://omics.informatics.indiana.edu/CRISPRone/
